# Inpatient management of type 2 Diabetes Mellitus: Does choice of insulin regimen really matter?

**Published:** 2014

**Authors:** Syed Tehseen Akhtar, Khalid Mahmood, Iftikhar Haider Naqvi, Aneel Sham Vaswani

**Affiliations:** 1 Dr. Syed Tehseen Akhtar, FCPS, Assistant Professor of Medicine, Dow University of Health Sciences, Karachi, Pakistan.; 2 Dr. Prof. Khalid Mahmood, FRCS, FCPS, Professor of Medicine, Dow University of Health Sciences, Karachi, Pakistan.; 3 Dr. Iftikhar Haider Naqvi, FCPS, Assistant Professor of Medicine, Dow University of Health Sciences, Karachi, Pakistan.; 4 Dr. Aneel Sham Vaswani, FCPS, Assistant Professor of Medicine, Dow University of Health Sciences, Karachi, Pakistan.

**Keywords:** Non-critically ill type 2 diabetic patients, Insulin regimen

## Abstract

***Objective: ***To assess inpatient management of non-critically ill type 2 diabetics with different insulin regimen.

***Methods: ***We reviewed the medical records of all non-critically ill type 2 diabetic patients more than 18 years of age in medical department of civil hospital Karachi and Dow University of Health Sciences from January 2011 to December 2012. We collected the data from case records in data collection sheets that fulfill the inclusion criteria and divided the study subjects into three groups according to insulin regimen they received.

***Results: ***A total of 416 patients were analyzed out of which 220 were male. Subjects were divided into three groups according to insulin regimen they received. Majority were put on sliding scale of insulin (44.7%), while 33.1% and 22.1% subjects received basal bolus and pre-mixed insulin regimen respectively. Patients treated with basal bolus regimen had greater improvement in glycaemic control with short duration of hospital stay as compared to other two groups. The mean hyperglycaemic events were higher in sliding scale group while mean hypoglycaemic events were higher in basal bolus group.

***Conclusion: ***In non-critically ill type 2 diabetic patients the basal bolus regimen is superior to sliding and pre-mixed insulin regimen. Sliding scale should be discouraged in non-critically ill type 2 diabetic patients.

## INTRODUCTION

High blood sugar levels in hospitalized patients with diabetes are connected with increased risk of complications. Better glucose control with insulin may improve clinical outcome and prevent the hospital complications. Uncontrolled blood sugar levels in hospitalized patients is associated with increased morbidity, mortality, and longer hospitalization, whereas optimal glycemic control results in better outcome.^1^^,^^3^ Therefore, it is imperative that blood glucose level in hyperglycemic patients should be properly controlled. However which insulin regimen should be preferred in non-critically ill Type 2 diabetics in medical ward is still being debated worldwide. In Pakistan it is common practice that physicians suggest sliding-scale of regular insulin in non-critically ill patients in medical wards. Sliding scale of regular insulin (SSI) is in use in the management of patients with diabetes since 1934.^4^ SSI is widely used in health care institutions^1^^,^^5^ because it is easy and convenient but it has the disadvantage of not delivering insulin in a physiologic manner, thereby leading to fluctuations in glycemic levels.^2^^,^^3^^,^^6^ Despite these drawbacks the use of SSI has been continued for almost 80 years.

Many studies including retrospective and prospective cohort studies^1^^,^^5^^-^^7^ have concluded that SSI should be discouraged because it has not been shown to be an effective means of achieving optimal glycaemic control in hospitalized patients. It is now recommended that hospitalized diabetic patients who are not critically ill should receive basal insulin along with scheduled preprandial doses of rapid-acting insulin and additional supplemental rapid-acting insulin to correct premeal .^8^

In most of the teaching hospital of Pakistan sliding scale of insulin is still used for its convenience and easy dosage. It is required to develop interventional and educational programs to improve inpatient and outpatient diabetes care. In order to assess inpatient management of hyperglycemia in non-critically ill type 2 diabetic patient we reviewed the medical records to determine the current state of glucose management in non-critically ill T2DM in medical ward of tertiary care hospital and compared the data with different regimen of insulin and its effect on glycemic control.

## METHODS

We reviewed the medical records of all patients older than 18 years of age, with a known history of type 2 diabetes, admitted in Medical wards of Civil Hospital and Dow University of Health Seciences Karachi during the past 2 years i.e. from January 2011 to December 2012. All information was obtained by chart reviews/case records. Patients included were:

- Males or females >18 years admitted to medical ward.

- Known history of type 2 diabetes mellitus more than 6 months, on diet control alone or taking any combination of oral antidiabetic agents (sulfonylureas, metformin, thiazolidinediones, DPP-4 inhibitors).

- Patients must had an admission blood glucose level more than 140 mg and less than 400 mg/dl without laboratory evidence of diabetic ketoacidosis (serum bicarbonate < 18 mEq/L or positive serum or urinary ketones).

We excluded those who had

- Type 1 diabetes.

- Hyperglycaemia without a known history of diabetes. 

- History of diabetic ketoacidosis and hyperosmolar hyperglycaemic state, or ketonuria. 

- Known HIV

 Critical medical or surgical illness is defined by American Association of Critical Care as those patients who are at high risk for actual or potential life-threatening health problems requiring intense and vigilant nursing care.^R^ It includes patients in intensive care unit or high dependency unit(ICU/HDU). 

- Clinically relevant hepatic disease or chronic kidney disease, as shown by a serum creatinine more than 2.5mg/dL.As CKD currently classified on the basis of e GFR which has a rough correlation with serum creatinine level. Creatinine level > 2.5 mg indicates stage 3 or more advance CKD eGFR 34.4 ml/L. 

- Diagnosed or suspected endocrine diseases like Cushing syndrome, Leprechaunism Lipodystrophic states, Wermer syndrome and Rabson-Mendenhall syndrome as they are associated with insulin resistance. 

- Pregnant or breast feeding female. 

 The data collection sheet included age, gender, weight, BMI, HbA1c, FBS, RBS, frequency of hypoglycaemic and hyperglycaemic events and duration of hospitalization and type of insulin regimen that is used. We divided the study subjects into three groups according to type of insulin regimen they were put on. These regimens were sliding scale, basal bolus and pre-mixed insulin regimen. In sliding scale adjusted dose of regular insulin in accordance with the results of preprandial blood glucose levels was used while in basal bolus multiple short acting insulin before each meal as bolus and intermediate insulin as basal at bed time were used. In pre-mixed 70/30 combination of regular and intermediate acting insulin is used twice daily.

Statistical analysis was performed on SPSS version 15. Continuous variable like age, weight, BMI, FBS, RBS, frequency of hypo and hyperglycaemic events and duration of hospitalization were expressed as mean ±SD. Discrete variables like gender, was expressed as percentage and proportion. One way ANOVA was applied and results of three insulin regimen was compared. A p-value <0.05 was considered statistically significant.

## RESULTS

During trwo years period, 477 diabetic patients were selected whereas 61 were excluded as they did not meet the inclusion criteria. Four hundred and sixteen patients were ultimately analyzed out of which 220(52.9%) were males. According to the insulin regimen subjects were divided into three groups, sliding-scale, basal bolus and pre-mixed 70/30 groups (Table-I). In sliding scale group number of patients was 186(44.7%), basal bolus group included 138(33.1%) subjects while number of patients in pre-mixed group was 92(22.1%). There were no significant differences in the mean age, BMI, admission blood glucose, or A1C between treatment groups. The mean hospital length of stay was 15.5 ±3.6 days in patients treated with sliding-scale, 7.8 ±1.9 days in the basal bolus treated group and 8.7±1.4 in pre-mixed group with p-value < 0.001.Patients treated with basal bolus insulin had greater improvement in glycaemic control than those treated with pre-mixed 70/30 and sliding scale group, but mean hypoglycaemic events were slightly higher in basal bolus groups (3.9±1) than other two treatment groups (2.9±0.8 and 3.1±0.8 in sliding-scale and pre-mixed group respectively). The mean hyperglycaemic events were higher in sliding scale group (9.4±6.9) than other two groups. (Table-II)

## DISCUSSION

In non-critically ill type 2 diabetic patients in medical wards glycaemic control remains unsatisfactory despite use of insulin. We reviewed the cases of non-critically ill inpatients with uncontrolled blood sugar level in medical ward of civil hospital Karachi and found that sliding scale of insulin is still number one choice of physician as 44.7% subjects of the study group were on this regimen similarly as in other academic institutions reported by Knecht^9^ and Schnipper.^10^

**Table-I:** Baseline clinical characteristics (n=416).

***Variable***	***Sliding scale***	***Basal Bolus***	***Pre-mixed 70/30***
No. of Patients (%)	186(44.7)	138(33.2)	92(22.1)
Age ± SD (Years)	52.3±7.1	49.8±9.0	52.7±11.2
Gender (%)			
Male	87(20.9)	95(22.8)	38(9.1)
Female	99(23.8)	43(10.3)	54(12.9)
Weight ± SD (Kg)	63.3±6.2	64.14±7.0	63.93±6.9
BMI ± SD (Kg/m2)	32±6	31±5	32±2

**Table-II:** Glycaemic control and duration of hospitalization with different insulin regimen

***Variable***	***Insulin Regimen***	***No. of patients***	***mean ±SD***	***95% Confidence Interval***		***p-value***
				***Upper***	***Lower***	
FBS	Sliding scale	186	154±24	150.69	157.77	<0.001
	Basal bolus	138	122±9	120.81	124.15	
	Pre-mixed70/30	92	133±9	131.29	135.25	
RBS	Sliding scale	186	273±40	267.28	279.03	<0.001
	Basal bolus	138	165±26	160.88	169.69	
	Pre-mixed70/30	92	190±21	185.90	194.62	
Duration of hospitalization	Sliding scale	186	15.5±3.6	14.99	16.03	<0.001
	Basal bolus	138	7.8±1.9	7.50	8.17	
	Pre-mixed70/30	92	8.7±1.4	8.44	9.04	
Hypoglycaemic events	Sliding scale	186	2.9±0.8	2.75	2.99	<0.001
	Basal bolus	138	3.9±1.0	3.71	4.06	
	Pre-mixed70/30	92	3.1±0.8	2.85	3.19	
Hyperglycaemic events	Sliding scale	186	9.4±6.9	8.36	10.36	<0.001
	Basal bolus	138	1.2±0.7	1.08	1.31	
	Pre-mixed70/30	92	1.7±0.8	1.51	1.88	

The over use of sliding-scale insulin (SSI) is because of convenience and simplicity and easy to implement and does not require to locate an attending physician concerning the necessary insulin dosage.^11^ Further there is fear of hypoglycaemia which encourage them to use SSI instead of basal bolus insulin.^12^ In our study group subjects on SSI regimen had more hyperglycaemic events and longer period of hospital stay than basal bolus regimen which is comparable with other studies which showed that use of SSI as the sole treatment for inpatient is ineffective and associated with several problems including hyperglycaemic events.^5^^,^^13^^,^^14^

 In this study we noticed that glycaemic control was significantly better in basal bolus regimen then SSI. In basal bolus regimen our patients got the NPH insulin instead of long acting glargine because of unavailability in Civil Hospital Karachi which is a public sector hospital. Control of fasting and random mean blood glucose level was superior with basal bolus regimen as compared to SSI and pre-mixed regimen. (Table-II). These findings are comparable with other studies.^15^^-^^17^ Umpierrez GE et al.^15^ had conveyed the results of a prospective, randomized multicenter trial in which they compared the basal-bolus insulin regimen with SSI and declared that the use of basal-bolus insulin produced greater improvement in blood glucose control than SSI alone. RABBIT 2 Surgery randomize study^17^ reported that basal-bolus regimen is associated with better glycemic control (66% vs 38%) and lower frequency of hospital complications than SSI, without increasing the number of severe hypoglycemic events.

Basal-bolus insulin is one of the most advanced approaches to diabetes care and offers a natural insulin delivery.^18^^,^^19^ The basal insulin deals with the glucose which is synthesized by liver, while the bolus insulin controls post meal glucose. Because of varying requirement of basal and bolus insulin in different people, this regimen is appropriate and fulfils the physiological needs of each individual**. **Fritsche A.^20^ reported that basal-bolus regimen using glargine/glulisine results in a significantly superior glycaemic control versus premix therapy in a population with long-standing insulin-treated T2DM. The above study also showed no increase in the rates of hypoglycaemia. Insulin therapy must provide both basal and nutritional components to achieve blood glucose targets. Hospitalized patients often require high insulin doses to achieve target glucose levels because of increased insulin resistance; thus, in addition to basal and nutritional insulin requirements, patients often require supplemental or correction insulin for the treatment. It is essential that patients and physicians should be aware of the need to achieve target blood glucose in order to reduce the morbidity and mortality associated with T2DM.^21^

## CONCLUSION

In non-critically ill diabetic patients the basal bolus regimen is superior to sliding and pre-mixed insulin regimen. Sliding scale should be discouraged in non-critically ill type 2 diabetic patients.

## Authors Contribution:

**STA:** Conceived, designed and did statistical analysis & editing of manuscript.

**ASV and IHN:** Did data collection and manuscript writing.

**KM:** Did review and final approval of manuscript.

**STA and IHN: **Take the responsibility and are accountable for all aspects of the work in ensuring that questions related to the accuracy or integrity of any part of the work are appropriately investigated and resolved.
